# Novel host plant use by a specialist insect depends on geographic variation in both the host and herbivore species

**DOI:** 10.1007/s00442-023-05490-y

**Published:** 2023-12-20

**Authors:** James P. Michielini, Xianfeng Yi, Leone M. Brown, Shan Ming Gao, Colin Orians, Elizabeth E. Crone

**Affiliations:** 1 Department of Biology, Tufts University, Medford, MA 02155 USA; 2 Present Address: Department of Evolution and Ecology, University of California, Davis, CA 95616 USA; 3 College of Life Science, Qufu Normal University, Qufu, China; 4 Present Address: Biology Department, James Madison University, Harrisonburg, VA 22807 USA; 5 Biology Department, Pomona College, Claremont, CA 91711 USA

**Keywords:** Plant–insect interactions, Oviposition, Introduced species, Euphydryas, Host choice

## Abstract

Understanding the circumstances under which insect herbivores will adopt a novel host plant is a longstanding question in basic and applied ecology. While geographic variation in host use can arise through differences in both herbivore preference and plant characteristics, there is a tendency to attribute geographic variation in host use to regional differences in herbivore preference alone. This is especially true for herbivores specialized to one or a few plant species. We compared how geographic variation in herbivore preference and host plant origin shape regional differences in host plant use by the specialized herbivore, *Euphydryas phaeton*. In parts of its range, *E. phaeton* uses only a native host, *Chelone glabra*, while in others, it also uses an introduced host, *Plantago lanceolata*. We offered female butterflies from each region the non-native host plant sourced from both regions and compared their oviposition behavior. The non-native host was almost universally rejected by butterflies in the region where only the native plant is used. In the region where butterflies use both hosts, females accepted non-native plants from their natal region twice as often as non-native plants from the other region where they are not used. Acceptance differed substantially among individual butterflies within regions but not among plants within regions. Thus, both individual preference and regional differences in both the insect and non-native host contributed to the geographic variation in different ways. These results highlight that, in addition to herbivore preference, regional differences in perceived plant suitability may be an important driver of diet breadth.

**Supplementary Information:**

The online version contains supplementary material available at 10.1007/s00442-023-05490-y.

## Introduction

Plant–herbivore interactions are ubiquitous across terrestrial ecosystems and many herbivores are highly specialized to one or a few hosts (Bernays and Chapman 1994; Forister et al. 2015). Despite having access to fewer resources relative to generalists, specialist herbivores persist due to their efficiency in host searching, consumption, and acquired defenses (Fox and Morrow 1981; Bernays 1992; Renwick 2001), enabling them to dominate communities at local scales (Sudta et al. 2022). Most specialist herbivores exhibit geographic variation in host use throughout their range but may specialize on a single host plant species at local scales (Fox and Morrow 1981). Ecologists and evolutionary biologists have long been interested in the mechanisms that drive variation in host diet breadth because of its relationship to diversification in both plants and insects (Mason 2016; Gompert et al. 2019; Kuussaari et al. 2000). Understanding how patterns of specialist herbivory vary in space and time is essential for understanding the evolution of plants and animals (Agrawal et al. 2012; Karban 1989), predicting future host shifts (Forister et al. 2013), and effectively managing both plant and animal species in changing environments (Braga and Janz 2021).

Regional differences in an insect species’ diet can depend on geographic variation in both insect preference and host plant traits. To date, numerous studies have documented the effects of variation in phytochemistry and morphological plant traits on insect host use (Jaenike 1990, Lawton and JH 1991; Renwick 2001; Clisshold et al. 2009; Harrison et al. 2016: Coley et al. 2018), but few have analyzed how variation in both herbivores and host plants contribute to geographic variation in host use. In one notable exception, Singer and Parmesan (1993) found that host plant oviposition preference by an oligotrophic herbivore, Edith’s checkerspot, *Euphydryas editha* (Nymphalidae), depends on the origin of both the butterfly and plant, i.e., butterflies were more likely to oviposit on some plant species if the plants were from sites where the butterflies used that plant species. More recent studies found that geographic variation in host use in the Melissa blue butterfly, *Lycaeides melissa* (Lycaenidae), is due to among-population differences in both insect preferences and host plant characteristics (Harrison et al. 2016; Gompert et al. 2019). Yet, studies that explicitly examine variation in both herbivores and host plants remain rare (e.g., Singer et al. 2002), implicitly suggesting that geographic variation in host plant use reflects geographic differences within herbivore species alone.

In this study, we evaluate contrasting patterns in the use of a non-native (novel) host plant by the Baltimore checkerspot, *Euphydryas phaeton*, and the extent to which this phenomenon can be explained by geographic differences within both the butterfly species and its non-native host plant. Unlike Edith’s checkerspot and the Melissa blue, the Baltimore checkerspot is a strict specialist in parts of its range, with oviposition and pre-diapause feeding almost exclusively restricted to its native host plant, white turtlehead *Chelone glabra* (Plantaginaceae). Half a century ago, Baltimore checkerspots in Eastern North America were first recorded ovipositing on a second, non-native plant, English plantain *Plantago lanceolata* (Plantaginaceae) (Stamp 1979). Some populations of Baltimore checkerspots in the northern part of their range now oviposit on English plantain and use it as a pre-diapause host instead of white turtlehead (Bowers et al. 1992b). During post-diapause development, Baltimore checkerspots will feed on English plantain throughout their range (Bowers et al. 1992b; Arriens et al. 2021), though it may be a lower-quality food plant in some respects (Abarca et al. 2019; Arriens et al. 2021; but see Brown et al. 2017; Muchoney et al. 2022).

In the southern part of their range, no populations of Baltimore checkerspot have been observed ovipositing on English plantain or using it as a pre-diapause host, despite the ubiquity of English plantain as a common lawn weed. Many traits of English plantain are known to differ among populations (Marshall et al. 2019), but there is relatively little spatial genetic structure in its introduced range (Smith et al. 2020). Given that the Baltimore checkerspot is a strict specialist and that English plantain has low genetic structure in its introduced range, it is credible that patterns of host use in the Baltimore checkerspot are driven solely by differences in insect preferences across its range. However, it is also credible that variation in both plants and insects contributes to variation in Baltimore checkerspot diet breadth. For example, as noted above, Singer and Parmesan (1993) saw evidence for both butterfly and plant variation affecting oviposition of *Euphydryas editha*, whereas (based on unpublished data mentioned in their discussion) Kuussari et al. (2000) did not find evidence for contributions of among-population host plant differences to oviposition preference of the related Glanville fritillary *Melitaea cinxia* (Nymphalidae).

We tested the relative contributions of host plants versus insect preference to geographic variation in host plant use, by comparing oviposition preference of butterflies from two regions for plants from those same two regions. We compared oviposition preference of female Baltimore checkerspots from Massachusetts, where English plantain and white turtlehead are both used, to oviposition preference of checkerspots from Maryland, where there are no reports of Baltimore checkerspot butterflies ever ovipositing on English plantain or feeding on it in the pre-diapause stage. In both regions, we offered English plantain from each region to gravid Baltimore checkerspot females to test whether they accepted each plant and attempted to oviposit. Specifically, we asked: (1) Do rates of oviposition acceptance of English plantain differ between butterflies in different regions? and (2) Do rates of oviposition acceptance of English plantain differ between plants from different regions? We also used these results to quantify the extent of phenotypic variation among individual insects and host plants within regions. Our results provide strong evidence that geographic variation in both the herbivore and the host plant contributes to geographic variation in oviposition behavior, even in this highly specialized system.

## Methods

### Study system

Baltimore checkerspots are a Nymphalid butterfly native to wetlands and wet meadows of Eastern North America. The Baltimore checkerspot is univoltine and lays eggs in clusters of several hundred in mid-summer. Caterpillars form silk nests after hatching and overwinter as fourth instar caterpillars, emerging in the subsequent spring (Bowers 1978). Pre-diapause caterpillars are highly specialized in their diet, feeding solely on the host plant on which they emerged (Bowers et al. 1992b). In contrast, post-diapause caterpillars become less specialized and can venture long distances from their original nest location, especially if their original plant source has been depleted (Bowers et al. 1992b).

English plantain, *Plantago lanceolata*, is a short-lived perennial forb, native to Eurasia, but naturalized in many locations worldwide (Smith et al. 2020). It is common in urban lawns and frequently mowed old fields, and often occurs on the margins of wet meadows in which the Baltimore checkerspots’ native host is found. Members of the genus *Plantago* are the native hosts of some butterflies in the Melitaeini tribe to which checkerspots belong, and *Plantago* species have been commonly adopted as an alternative host by North American checkerspot species (Singer et al. 2008). *Euphydryas phaeton* as well as the variable checkerspot *E. chalcedona,* and Edith’s checkerspot *E. editha* are known to feed on English plantain (Stamp 1979; Graves & Shapiro 2003; Schultz et al. 2016). The earliest record of adoption of English plantain in any of these species was in a population of Edith’s checkerspot in 1953 in Nevada (Singer et al. 2008). English plantain was apparently ‘very common’ in Mid-Atlantic states, including Maryland, by the early nineteenth century (Rafinesque Schmaltz 1811; Mack 2003). In the 1970s, Baltimore checkerspots in the northeastern US (Massachusetts, New York and Rhode Island) were frequently reported ovipositing on English plantain, using it as a primary pre-diapause food source (Stamp 1979; Bowers et al. 1992b). To our knowledge, this behavior is not present in populations south of New York.

English plantain is known to have considerable intraspecific variation across a variety of traits and is therefore an ideal candidate to explore how variation in plants may affect the species’ interactions. Intraspecific differences of English plantain have been extensively studied in a phytochemical, structural, and population genetic context. English plantain has extensive within-population variation in defense-related traits, such as the concentration and the absolute amount of iridoid glycosides (Bowers et al. 1992b; Darrow and Bowers 1997). Iridoid glycosides are toxic to most generalist species as they target proteins during digestion (see Dobler et al. 2011). Baltimore checkerspots are stimulated to feed by the presence of the iridoid glycosides, aucubin and catalpol (Bowers 1983), and sequester these chemicals which confer protection against viral pathogens (Muchoney et al. 2022; but see Laurentz et al. 2012 which found no strong relationship between iridoid glycoside concentration and pathogen defense in the related *Melitaea cinxia*) and against predators (Bowers 1980). Other traits, such as thermal tolerance, vary geographically across the native range of English plantain (Marshall et al. 2019). Populations in the native European range of English plantain also have strong spatial genetic structure associated with geographic distance and precipitation seasonality, although this variation tends to be lower in the nonnative ranges (Smith et al. 2020).

### Experimental design

We evaluated oviposition preference of female checkerspot butterflies at one field site in Massachusetts and two in Maryland in the summer of 2018. The study was carried out from June to July, just after the emergence of butterflies at our sites in each state. Experimental trials in Maryland took place at two sites: Alesia (39.7, − 76.8) and White Hall (39.7, − 76.6) from June 6th to June 19th. Alesia and White Hall sites were both wet meadows where butterflies used white turtlehead as a pre-diapause host plant and added additional plant species, including English plantain, as post-diapause hosts (Arriens et al. 2021). Experimental trials in Massachusetts took place in Upton (42.2, − 71.7) where English plantain is abundant and used as a pre- and post-diapause host. White turtlehead is rare at the Upton site and we have only occasionally observed caterpillar nests on white turtlehead there. Experimental trials in Massachusetts took place from July 2nd to July 9th. Trials were carried out on sunny days between 11:00 AM and 5:00 PM local time while butterflies were flying. A total of 18 butterflies were tested (9 in each state) across a total of 248 trials on English plantain (average 13.7 trials per butterfly). Experiments were conducted in the field because Baltimore checkerspots in Maryland have small populations that may be in decline (Frye 2013), and we did not want to remove individuals from these sensitive populations. Each butterfly was given a unique, four-color identification code using metallic gel pens to ensure they were not tested twice.

**Fig. 1:**
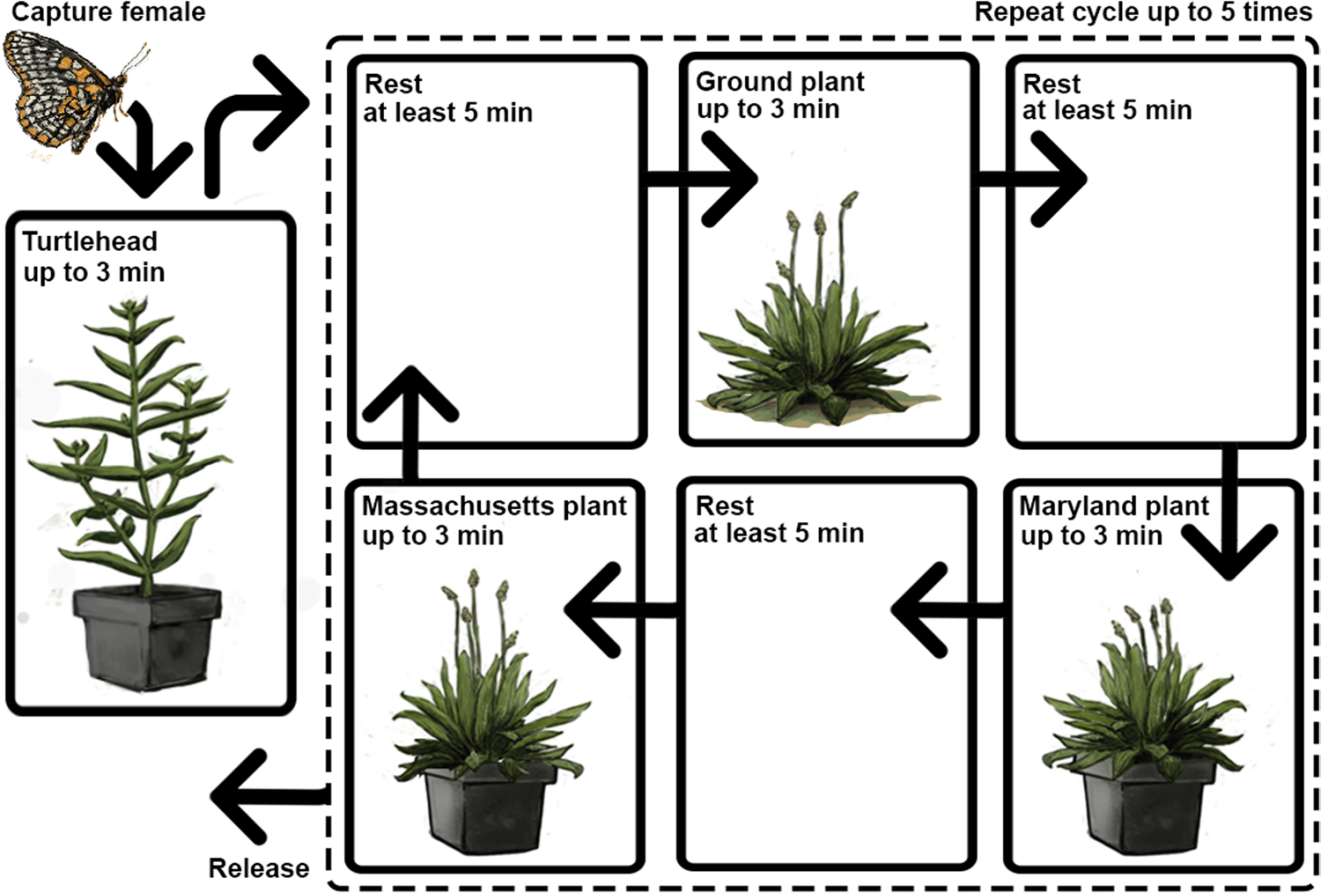
Experimental design to measuring Baltimore checkerspot host plant acceptance (adapted from Singer 1982). Captured females were marked and placed in a tent on a potted white turtlehead plant. If a female attempted to oviposit within the first three minutes, it was then used in trials with English plantain. After resting for five minutes the female was then placed on a randomly selected English plantain and observed until they attempted to oviposit or until three minutes passed, which we considered a rejection of the plant. A single cycle of trials included three English plantain plants: one potted plant from Maryland, one potted plant from Massachusetts, and one “ground” plant growing naturally on site. Females were allowed to rest in between offerings with the plant at least five minutes. This cycle was repeated up to five times in total using randomly selected plants from different sites when offering potted plants, after which females were released. Each female willing to oviposit on white turtlehead was therefore tested on 15 different English plantain plants altogether and females were not tested more than once

Unlike many butterflies which lay eggs singly on host plants, Baltimore checkerspots lay egg clusters of up to hundreds of eggs. This trait introduces experimental challenges in terms of evaluating preference and willingness to use a host plant. Our experimental setup (Fig. 1) was based on Singer’s study of oviposition preference in Edith’s checkerspot *Euphydryas editha* (1982). Mated females were caught and placed on the native host plant, white turtlehead, and observed for three minutes to assess their willingness to oviposit. Those that visually appeared to be mated and performed typical oviposition behavior, tapping the underside of a leaf using their forelimbs and curling their abdomen, were considered gravid and used for English plantain preference tests while those that did not were released. During preference trials, female butterflies were rotated among single English plantain plants from three sources: potted plants from Maryland, potted plants from Massachusetts, and naturally growing plants. For each trial, butterflies were placed on an English plantain plant within a net cage and monitored for up to three minutes to see if they began to exhibit oviposition behavior. If the female did begin to exhibit oviposition behavior on the plant, the butterfly was removed before laying any eggs. Between trials, butterflies were allowed to rest in a cage with no host plants for at least five minutes. The sequence of the individual plants that were used in trials was selected from a randomly generated list. Butterflies were released after completing five cycles of trials wherein three new randomly selected plants, one from each of the three sources, were used (Fig. 1). Butterflies were tested for a total of approximately 2.5 h across all trials including time while they were at rest. On occasion, trials would not be fully completed due to weather or time constraints.

Local lineages of white turtlehead plants were used in both Maryland and Massachusetts as a control to test for gravidity of females. White turtlehead plants were obtained from two nurseries, Chesapeake Natives in Maryland and Garden in the Woods in Massachusetts, which carry local genotypes. English plantain, *Plantago lanceolata*, was sourced from three sites in each study region (Maryland and Massachusetts). In Maryland, plants were collected from Alesia and White Hall, where experimental trials were conducted, and from a third location in Norrisville (39.7, 76.5). In Massachusetts, English plantain was collected from Upton, where the experimental trials were conducted, and from Sharon (42.1, − 71.2) and Harvard (42.5, − 71.6). We collected plants from these sites because they were butterfly habitat where checkerspot populations were present or formerly present in recent years. These plants were collected and potted approximately one week before trials were conducted in each state. Potted plants were watered daily and were kept outside, exposed to natural sunlight. As an experimental control for effects of potting, haphazardly selected English plantain growing naturally in the soil at each site was also included in oviposition trials; hereafter we refer to these experimental controls as “ground” plants. For potted plants, we used ten English plantain plants from each site; we used a separate set of plants for trials in Massachusetts and for trials in Maryland, for a total of 120 potted plants. Because plants were chosen at random for each trial, not all were used. We also avoided using plants that showed obvious signs of damage. A total of 139 English plantain plants were used in the experiment, including both potted and ground plants. All plants used were approximately similar in height, size, and number of leaves.

### Data analysis

All analyses were carried out in R (version 4.1.3, R Core Team 2022). Generalized linear models (GLMs) were implemented with the glm() function, and generalized linear mixed models (GLMMs) were implemented with the lme4::glmer function (Bates et al. 2015). For all models, we evaluated statistical significance of terms using type II marginal likelihood ratio tests, implemented with the car::Anova function (Fox and Weisberg 2019), which removes terms from the model individually and evaluates whether removal improves model fit. We report whether each term significantly improved the model fit and the associated chi-squared statistics from the likelihood ratio test (see Supplemental Table 1 for complete table with all model specifications and results).

Butterfly response to any plant was recorded as either an acceptance (oviposition behavior), or rejection (no oviposition behavior). In our main analysis, we tested the effects of geographic differences between plants and butterflies using a binomial family, logit link, GLMM with a fixed effect of butterfly state origin (MA or MD), plant state origin (MA or MD), and their interaction, as well as random effects of butterfly individual, plant individual, and plant site origin. Butterfly individual and plant individual were included as random effects to account for individual variation since each butterfly was offered multiple plants, and potted plants were used with different butterflies. In addition to statistically accounting for repeated measures of individuals, these random effects have an ecological interpretation; they are estimates of how much individuals vary within populations of each species (e.g., see Brown and Crone 2016). For similar reasons, the random effect of plant site accounts for differences among sites within a region that affect the acceptability of plants, and the variance associated with the random effect of site is a measure of among-site differences in acceptability of plants. However, because including plant ID did not explain any of the variation in oviposition preference (see *Results*) and occasionally prevented model convergence, we excluded this term from models used for inference about geographic differences in host quality and oviposition preference.

As a secondary test for preference, we analyzed whether acceptance of English plantain changed through time, both within trials and across the season. We conducted this test because the threshold for oviposition acceptance among insects is widely known to decrease over periods of deprivation (Singer 1982; Miller & Strickler 1984). We implemented these tests by repeating analyses twice, once with an additional fixed effect of the ordinal position of each plant within a trial and once with an additional fixed effect of day of year. These analyses were only conducted for butterflies from Massachusetts, since there was essentially no variation in oviposition acceptance of English plantain in Maryland (see *Results*).

We also compared acceptance rates for our two procedural controls: (1) oviposition acceptance of white turtlehead in Maryland versus Massachusetts trials (as a coarse measure of regional differences in preference for white turtlehead), and (2) oviposition preference of potted versus ground English plantain plants (as an experimental control for effects of using potted plants). We compared rates of acceptance of white turtlehead using simple binomial family, logit link GLMs, with region (Massachusetts versus Maryland) as a fixed effect. We compared acceptance of potted plants from each region to “ground” plants in each state (i.e., we compared potted Maryland plants tested in Maryland versus ground plants in Maryland, and potted Massachusetts plants tested in Massachusetts versus ground plants in Massachusetts). These comparisons used a binomial family, logit link, GLM with fixed effects of state (Maryland versus Massachusetts), plant type (potted or natural), and their interaction. Random effects of plant ID and site were removed due to convergence issues.

As a second metric of acceptance, we analyzed time until oviposition for butterflies willing to oviposit. This analysis included only trials in which butterflies demonstrated oviposition behavior on the non-native host, and therefore included only butterflies from Massachusetts (see *Results*). Time to oviposition was analyzed using a Gaussian (normal) GLMM with log-transformed time to oviposition behavior as the response variable, a fixed effect of plant state of origin as the predictor variable, and random effects of plant individual, butterfly individual, and plant site of origin. As in the analyses of acceptance, plant site did not explain any of the variation in time to oviposition (see Results) and occasionally prevented model convergence. We therefore excluded this term from the final models.

## Results

**Fig. 2:**
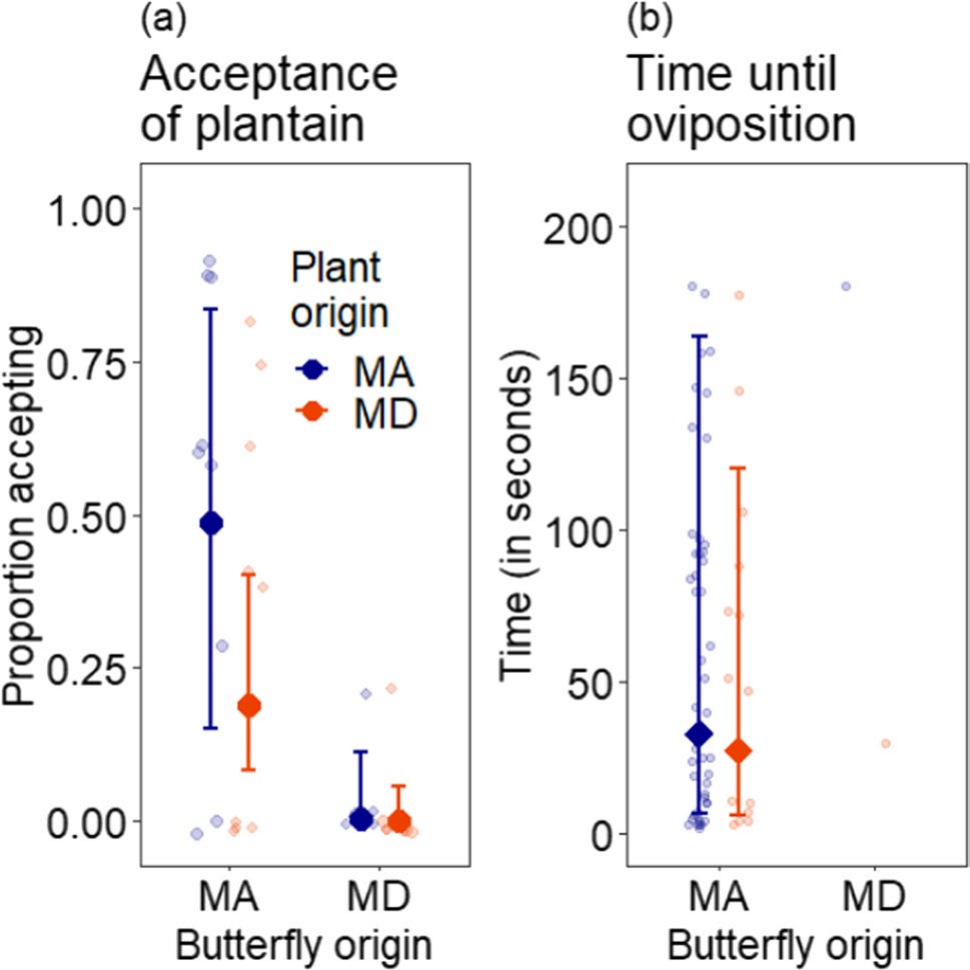
**a** Estimated proportion of butterflies accepting English plantain given the state origin of both the butterfly and the plant (MA = Massachusetts, MD = Maryland). Points represent the proportion of plants expected to be accepted from the main analysis model. Error bars represent 95% confidence intervals using standard errors. Smaller transparent points represent the raw data average proportion of plants accepted by each butterfly with sizes proportional to the number of trials carried out with that butterfly and the plants of the given state (ground and potted plants are grouped and therefore plants and butterflies from the same state are overrepresented). *N* = 248 across 18 butterflies. b. Estimated time (in seconds) until female Baltimore checkerspots began to exhibit oviposition behavior on English plantain for Massachusetts butterflies. Error bars represent 95% confidence intervals using standard errors. *N* = 61 across 7 butterflies. Smaller transparent points represent the times until attempted oviposition for each trial (rejections are not plotted here)

Overall, acceptance of English plantain differed significantly with butterfly state of origin (*χ*^2^ = 9.4, *df* = 1, *P* = 0.002) and plant state of origin (*χ*^2^ = 7.0, *df* = 1, *P* = 0.008; Fig. 2a). Gravid females from Massachusetts were more likely to accept English plantain (acceptance probability 0.3, CI = 0.12–0.62), than gravid females from Maryland (acceptance probability 0.002, CI = 0.0001–0.08). Gravid females from Massachusetts accepted Massachusetts English plantain about half the time (acceptance probability 0.49, CI = 0.15–0.84) and Maryland English plantain about one-fifth the time (acceptance probability 0.19, CI = 0.083–0.40), reflecting the main effects of plant state of origin. Only one of the nine gravid females from Maryland attempted to oviposit on English plantain; out of ten trials on potted English plantain plants, this butterfly attempted to oviposit twice, once on a plant from each state. The proportion of acceptance of plantain by females was not significantly affected by the interaction between butterfly origin and plant origin (*χ*^2^ = 0.56, *df* = 1, *P* = 0.45). Overall, random effects of butterfly identity explained substantial variation among female butterflies (standard deviation (SD) = 2.31). Random effects of plant identity and plant site of origin did not explain any substantial variation in terms of oviposition acceptance (SD < 0.001 for both terms).

Oviposition behavior did not change as a function of plant sequence within trials (GLMM with added effect of plant’s sequence position in trial: slope: − 0.005, standard error = 0.007, *χ*^2^ = 0.598, *df* = 1, *P* = 0.44). However, Massachusetts butterflies were more likely to accept English plantain later in the breeding season (GLMM with added effect of day since beginning of trials: slope: 0.17, standard error = 0.08, *χ*^2^ = 11.75, df = 1, *P* < 0.001). There was no interaction between day of year and state of origin of the plant (*χ*^2^ = 1.16, df = 1*, P* = 0.28), meaning butterflies increased acceptance of plants from both states at similar rates.

Time to oviposition was only analyzed for butterflies from Massachusetts (Fig. 2b), since only one butterfly from Maryland ever attempted to oviposit on English plantain. For trials where the butterfly did exhibit oviposition behavior, time until oviposition when placed on English plantain did not differ as a function of plant state of origin (*χ*^2^ = 0.082, d*f* = 1, *P* = 0.77). As in the analyses of oviposition acceptance, oviposition time differed somewhat among individual butterflies (random effect SD = 33.88). Unlike the analysis of oviposition acceptance, there was also some difference among individual plants (random effect SD = 34.78).

**Fig. 3:**
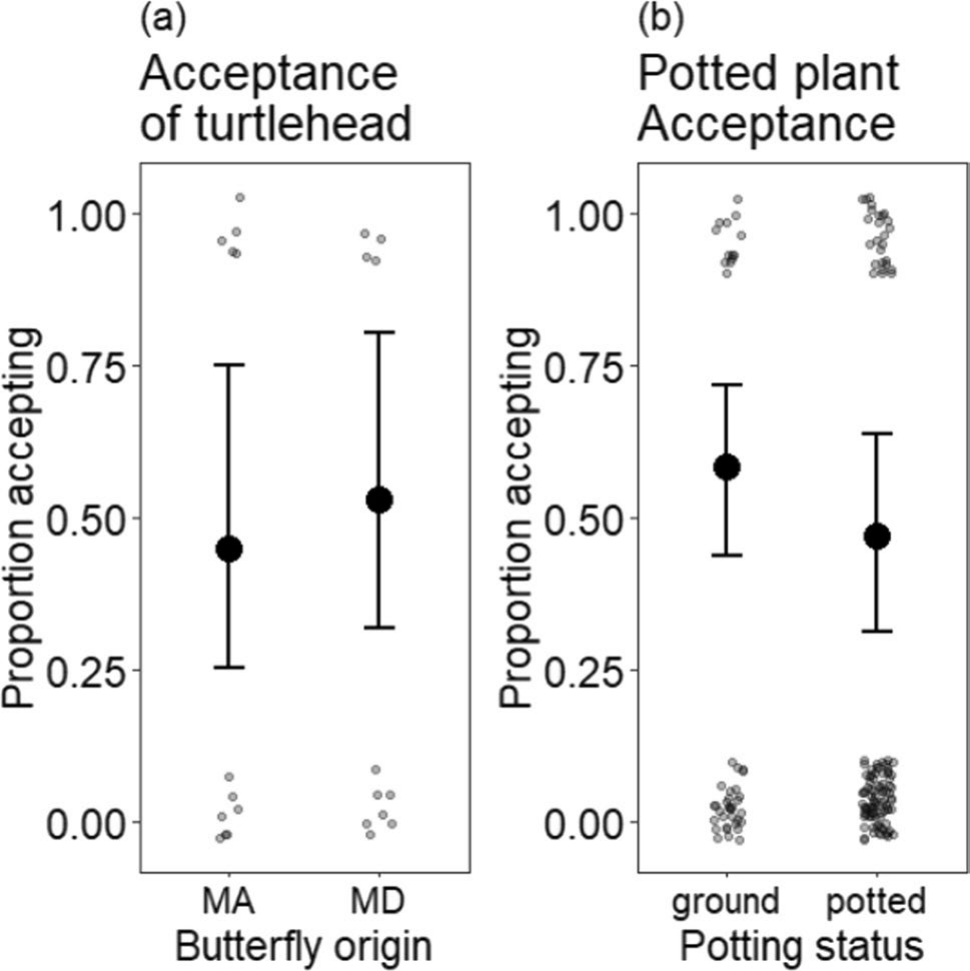
**a** Baltimore checkerspot female estimated willingness to oviposit on white turtlehead given the butterfly state of origin in preliminary control trials for gravidity. Error bars show 95% confidence intervals using standard errors. Smaller transparent points represent the raw data for each preliminary trial with white turtlehead. *N* = 37 (20 butterflies in Massachusetts and 17 butterflies in Maryland). **b** Baltimore checkerspot female estimated willingness to oviposit on English plantain if potted in control trials. Points represent the probability a female would accept the plant if it were potted or growing naturally in soil at the site where the trail was conducted and where the female was captured. Smaller transparent points represent the raw data for all trials in the main analysis. Error bars show 95% confidence intervals using standard errors. *N* = 159 across 18 butterflies

Our experimental controls supported inference from these experiments. We evaluated 17 Baltimore checkerspot butterflies in Maryland and 20 Baltimore checkerspot butterflies in Massachusetts on the native host plant, white turtlehead. In both states, nine of the butterflies exhibited oviposition behavior on white turtlehead (Fig. 3a). The proportion of butterflies willing to oviposit on white turtlehead did not differ between Maryland and Massachusetts (*χ*^2^ = 0.23, df = 1, *P* = 0.63; estimated probabilities of white turtlehead acceptance in Maryland: 0.58, CI: 0.4–0.84; and Massachusetts: 0.45, CI: 0.2–0.638). In other words, there were no significant differences in oviposition acceptance of the native white turtlehead between the two states.

In the comparison of potted plants versus plants in the ground in each state, butterflies had no significant preference for either potted or naturally growing English plantain (χ^2^ = 1.4, df = 1, *P* = 0.24) and there was no interaction between state and potting status (*χ*^2^ < 0.001, df = 1, *P* = 1). The nonsignificant effect was in the direction of slight preference for naturally growing ground plants over potted plants (Fig. 3b). As in the main analysis of data from potted plants only, this analysis showed strong differences among states (*χ*^2^ = 8.25, df = 1*, P* = 0.004). The same seven butterflies that attempted to oviposit at least once on potted plants in Massachusetts also attempted to oviposit on plants in the ground. The butterfly from Maryland that did attempt to oviposit did so only on potted plants (one from Massachusetts and one from Maryland).

## Discussion

The geographic disparity in the interaction between Baltimore checkerspots and English plantain reflects variation in both interacting species. Butterflies from Massachusetts were more likely than butterflies from Maryland to accept English plantain, and plants from Massachusetts were preferred over plants from Maryland. However, the patterns of regional variation differed qualitatively between both members of the interaction with butterfly origin having a larger effect than plant origin (See Fig. 2 and Results). The patterns found for the specialist Baltimore checkerspot broadly resemble geographic variation in the oligotrophic Edith’s checkerspot (Singer and Parmesan 1993) in two ways. First, populations of Edith’s checkerspots which did not use one host plant species in the field nearly always rejected it in oviposition trials (similar to Baltimore checkerspots and English plantain in Maryland). Second, another Edith’s checkerspot population preferred plants from a site where they were used in the field over plants from sites where they were not used (similar to Baltimore checkerspots and English plantain in Massachusetts). Our results differ from populations of the Melissa blue, in which variation in oviposition preference for alfalfa (*Medicago sativa*) depended on both plant and butterfly population of origin but did not align with host use in the field (Harrison et al. 2016).

The significant effect of plant origin on oviposition acceptance and the minimal effect of plant identity or site of origin on oviposition acceptance suggests that the perceived quality of a host plant is determined at regional scales. The two regions from which plants were collected were separated by approximately 500 km, but we found no evidence for differences among sites within regions which were no more than 60 km apart. By comparison, the populations of Edith’s checkerspots which exhibited similar patterns (Singer and Parmesan 1993) were approximately 190 km apart. Patterns of host use in Edith’s checkerspot are more variable across California, however, with females ovipositing on different hosts in nearby sites or even within a metapopulation (Singer and Wee 2005). Similarly, there is no clear geographic boundary between Melissa blue populations that feed on non-native alfalfa are those that do not (Forister et al. 2013; Harrison et al. 2016). Unlike these species, it appears that Baltimore checkerspot populations willing to utilize the novel host are geographically separated at higher latitudes. It would be worth investigating patterns of host use more broadly in the species, especially because Baltimore checkerspots use different native hosts in some parts of its range in Michigan and Missouri (Scholtens 1991).

Given the limited genetic population structure observed in its introduced range (Smith et al. 2020), it is possible that plastic differences driven by climate or phenology drive the regional discrepancy in the acceptability of English plantain. One possible explanation for geographic variation in host plant acceptance is climate-induced variation in phytochemistry. Baltimore checkerspot feeding is known to respond to variation in two iridoid glycosides: catalpol and aucubin (Bowers et al. 1992a). Both chemicals act as feeding stimulants, and Baltimore checkerspots sequester them as chemical defenses (Bowers and Puttick 1986). Baltimore checkerspots have higher concentrations of catalpol and almost no aucubin when reared on white turtlehead after diapause and have higher concentrations of aucubin when reared on English plantain after diapause (Muchoney et al. 2022). In English plantain, both of these secondary chemicals are also known to vary with climate. For example, Darrow and Bowers (1997) reported an elevational gradient in catalpol concentrations of English plantain, with more catalpol in plants from higher-elevation sites. Orians et al. (2019) exposed English plantain plants to drought and heat treatments, with warmer, drier conditions resulting in more aucubin and less catalpol. From these studies, is tempting to speculate that English plantain from the cooler northern location, Massachusetts, have a higher concentration of catalpol, and chemical profiles more similar to white turtlehead than English plantain plants from Maryland. Regional phenological differences and the age of plants used in the experiment may have also elicited the disparity in oviposition acceptance in Maryland and Massachusetts plants since plants were collected at similar times prior to the start of the trials. English plantain from Massachusetts could have been slightly younger and therefore may have had lower concentrations of catalpol (Jarzomski et al. 2000). The buckeye butterfly *Junonia coenia* (Nymphalidae), which also feeds on *P. lanceolata*, is known to oviposit more often on younger, less well-defended plantain (Quintero et al. 2014). Potential differences in both phytochemistry and phenology and their effects on oviposition preference between these regions merit further investigation.

More generally, English plantain has considerable within- and among-population genetic and plastic variation (Marshall et al. 2019; Smith et al. 2020). In English plantain, secondary chemistry has a genetic basis that can interact with environment to affect iridoid glycoside content (Bowers et al. 1992b). Concentrations of both catalpol and aucubin were higher in family lines collected from semi-natural field than a mowed lawn (Adler et al. 1995). Darrow and Bowers (1997) also reported lower aucubin and catalpol concentrations in English plantain from mowed sites, though they did not evaluate whether this difference was due to heritable variation. In our study regions, geographic variation in land management might mimic some of the differences between mowed lawns and more natural areas. Sites in Massachusetts were typically small meadows in natural areas managed for conservation and light recreation. Sites in Maryland were typically small meadows in the corners of farm fields that were too wet to farm. We used sites with different land use patterns because, to our knowledge, there were no natural areas managed for Baltimore checkerspots in our study region in Maryland that were occupied by Baltimore checkerspots at the time of our study.

Adoption of novel hosts has been observed multiple times in butterflies (Yoon and Read 2016) and especially in the Melitaeini tribe to which checkerspots belong (Thomas et al. 1987; Singer and Wee 2005). In some cases, novel host plants are ultimately preferred over native hosts. We did not explicitly test for geographic variation in preference for the native host plant, and our trials on English plantain included only butterflies who were willing to oviposit on the native white turtlehead. In part, this design was because no previous study of Baltimore checkerspots specifically have revealed a preference for exotic English plantain over native white turtlehead when white turtlehead is available (Bowers 1992b). However, some populations of Baltimore checkerspots in Massachusetts and adjacent states appear to exclusively use English plantain (Bowers and Richardson 2013). Toward the end of our study, there were three Massachusetts butterflies that did not accept white turtlehead but were still offered English plantain. These butterflies were from the Upton site in Massachusetts, where white turtlehead is rare. These butterflies were not included in our main analyses, but two of these three individuals demonstrated oviposition acceptance behavior when placed on English plantain. To further explore possible preference for English plantain over white turtlehead, we conducted a post hoc analysis comparing time to acceptance of white turtlehead versus English plantain, using female Massachusetts butterflies that were willing to oviposit on both hostplant species (Supplemental Fig. 1). In this analysis, time to oviposition acceptance did not differ between white turtlehead and English plantain (post hoc GLMM of time to acceptance, with a main effect of plant species and random effect of butterfly ID: *χ*^2^ = 0, df = 1, *P* = 0.99). Nevertheless, it would be interesting to more explicitly test whether some northern populations of Baltimore checkerspot butterflies have evolved preference for English plantain over white turtlehead.

Baltimore checkerspot populations seem to be declining in Maryland (Frye 2013) but are stable in more northern parts of their range, including Massachusetts (Breed et al. 2013; Michielini et al. 2021). It is interesting to contemplate whether population viability would increase or decrease in Maryland if Baltimore checkerspots were willing to oviposit on English plantain in Maryland. In Maryland, post-diapause larvae of Baltimore checkerspots prefer white turtlehead and grow faster on white turtlehead (Arriens et al. 2021). In addition, Abarca et al. (2019) reported that Baltimore checkerspot butterflies collected in Massachusetts were less likely to survive heatwaves if reared to diapause on English plantain (sourced from Washington D.C.; Abarca *pers. comm.*) than if reared on white turtlehead. It would be interesting to repeat Abarca et al.’s experiment using English plantain from both regions, given the differences in preference and potential chemical differences between English plantain from Maryland versus Massachusetts. Even if larval survival is lower on English plantain at some life stages, however, English plantain seems to be a suitable host plant for Baltimore checkerspots in Massachusetts in terms of population persistence (Brown et al. 2017). This may be because the spatial structure and arrangement of English plantain on the landscape supports substantially larger pre-diapause nest sizes than does white turtlehead (Brown et al. 2017; L. M. Brown, *pers. obs.*). This difference is challenging to account for in laboratory-based studies, and the larger per nest number of individuals supported by English plantain in the field could counter lower survival at the individual level seen in the laboratory.

Studies comparing butterfly host plant preferences among host plant species often use plants sampled from a single source population (Wehling & Thomspon 1997; Ladner and Altizer 2005; Haan et al. 2021; McCarty and Sotka 2013; c.f. Singer et al. 2002). In some cases, the results of these studies might change if there were substantial within-species variation among host plant populations. For example, Wehling and Thompson (1997) evaluated Anise swallowtail (*Papilio zelicaon*) preference by offering plants sourced from a single area to butterflies sourced from populations across their geographic range and found that rank order preference for hosts was largely conserved across different sites. If apparent host quality differed among populations at levels comparable to those we observed between plants from different regions, however, the rank order of Anise swallowtail hosts may not be as widely conserved. For example, a doubling of preference (similar to the difference in acceptance of English plantain between states by Massachusetts’ Baltimore checkerspots) for a lesser-used plant *Foeniculum*, could have changed the rank order preference of some Anise swallowtail populations. Ladner and Altizer (2005) found similar results in monarch butterflies: both eastern and western monarchs (*Danaus plexippus*) preferred swamp milkweed (*Asclepias incarnata*) over other milkweed species. In this case, monarch butterflies from western and eastern North America both laid the majority of eggs on swamp milkweed when presented with an array of milkweed species (Ladner and Altizer 2005); a doubling of preference for any other milkweed species would not change this rank order. In general, however, it would be useful to know more about how geographic variation in host plants affects butterfly oviposition preference.

Our results emphasize the importance of variation in both insects and host plants in determining intraspecific variation in host plant preference, even in a narrowly specialized species like the Baltimore checkerspot butterfly. In our system, these differences in oviposition acceptance of a non-native host plant appear to reflect different causal factors. High among-individual variation in butterflies is broadly consistent with a genetic basis for variation in preference among individuals. Heritable differences in host plant preference are common in other checkerspot species (Singer et al. 1994; Kuussaari et al. 2000), and early studies of Baltimore checkerspots (Brussard and Vawter 1975) suggested high within-population genetic diversity. Revisiting host plant preference with modern genomic methods could be an interesting next step in understanding this system. In contrast, low among-individual variation in plant quality, as measured by oviposition acceptance, seems more consistent with responses to regional climate differences, though there could of course be fixed genetic differences among regions. It is tempting to speculate that, if English plantain plants in Maryland were more appealing to Baltimore checkerspots, a host range expansion to this widespread weedy plant could help declining checkerspot populations persist in this part of their range. On the other hand, it may be that English plantain is a lower-quality host plant in Maryland than Massachusetts in terms of larval performance (e.g., Abarca et al. 2019), and a shift in preference could negatively impact population viability. In either case, understanding the future dynamics of this system will depend on understanding the basis of variation in both players in the interaction.

### Supplementary Information

Below is the link to the electronic supplementary material.

Supplementary file1 (DOCX 35 KB)

## Data Availability

Both the data used in these analyses and the R file used for this analysis are archived in the Dryad data repository: 10.5061/dryad.1vhhmgr02.

## Code availability

Should the manuscript be accepted, the data used for these analyses will be archived in a public repository and the data DOI will be included in the article.
