# Supplementary material for: Novel host plant use by a specialist insect depends on geographic variation in both the host and herbivore species
Source: Oecologia. 2023 Dec 20;204(1):95–105. doi: 10.1007/s00442-023-05490-y (PMC10830605; doi:10.1007/s00442-023-05490-y)
Supplement: Supplementary file 1 — Supplementary file1 (DOCX 35 KB) [file 442_2023_5490_MOESM1_ESM.docx]

**Novel host plant use by a specialist insect depends on geographic variation in both species**

**Authors:** Michielini, James P.^1,2^; Yi, Xianfeng^3^; Brown, Leone M^1,4^; Gao, Shan Ming^5^; Orians, Colin^1^; Crone, Elizabeth E.^1,2^

**Author Affiliations:** 1. Department of Biology, Tufts University, Medford, MA USA 02155, 2. Current address: Department of Evolution and Ecology, University of California, Davis, Davis CA USA 95616, 3. College of Life Science, Qufu Normal University, Qufu China, 4. Current address: Biology Department, James Madison University, Harrisonburg VA USA 22807, 5. Biology Department, Pomona College, Claremont CA USA 91711

**Corresponding Author:** James Michielini

Email: [jpmichielini@ucdavis.edu](mailto:jpmichielini@ucdavis.edu)

Telephone: 516-306-7377

Fax: (530) 752-1449


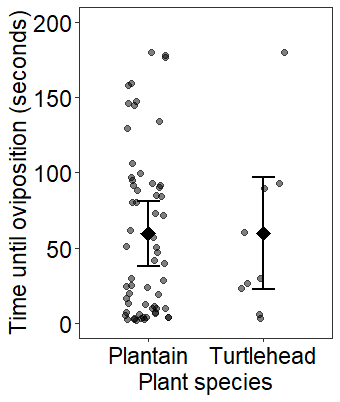


**Supplemental Fig. 1** The estimated time (in seconds) until oviposition behavior was observed for Massachusetts Baltimore checkerspot females for all trials on English plantain, and on white turtlehead where the female accepted the plant (i.e., any instances where females did not attempt to oviposit are excluded). Points indicate the amount of time until the female attempted to oviposit and diamonds the estimated average time to oviposition behavior from the model examining differences between the species. Points are jittered and opaque for visibility; more points are present for English plantain because individual females were given multiple English plantain plants while each butterfly was only ever tested once on white turtlehead (see Methods & Fig. 1 for more information). Error bars show 95% confidence intervals. N = 70 (61 English plantain and 9 white turtlehead).

**Supplemental Table 1.** All models are included in the main text. Each model is tabulated independently with the R code specifications provided. Fixed and random effect terms along with their relevant information are provided below the model specifications.

| **English plantain acceptance model:**  lme4::glmer(Acceptance ~ Butterfly state of origin * Plant state of origin + (1 \| Butterfly ID) +  (1 \| Plant ID^✝^) + (1 \| Plant Site), family = binomial(link = “logit”))  ^✝^Plant ID was removed from the final model due to low explanatory power and occasionally preventing convergence; this did not change the results of the model for the terms that remained included. | | | | | | | |
| --- | --- | --- | --- | --- | --- | --- | --- |
| **Fixed effect term** | **Coefficient** | **St. error** | **χ^2^** | **df** | **P** | **Random effect term** | **σ** |
| Intercept | -0.05 | 0.85 |  |  |  | Plant ID^✝^ | <0.001 |
| Butterfly State (MD) | -5.78 | 1.89 | 9.42 | 1 | 0.002 | Butterfly ID | 2.31 |
| Plant State (MD) | -1.40 | 0.51 | 7.05 | 1 | 0.008 | Plant Site | <0.001 |
| Interaction Term | 1.20 | 1.61 | 0.56 | 1 | 0.453 |  |  |
| **Time to oviposition model:**  lme4::glmer(Time ~ Plant state of origin + (1 \| Butterfly ID) + (1 \| Plant ID) + (1 \| Plant Site^✝✝^),  family = gaussian(link = “log”))  ^✝✝^Plant Site was removed from the final model due to convergence issues | | | | | | | |
| **Fixed effect term** | **Coefficient** | **St. error** | **χ^2^** | **df** | **P** | **Random effect term** | **σ** |
| Intercept | 3.74 | 0.68 |  |  |  | Plant ID | 34.8 |
| Plant State (MD) | -0.17 | 0.59 | 0.08 | 1 | 0.774 | Butterfly ID | 33.9 |
|  |  |  |  |  |  | Plant Site^✝✝^ |  |
| **Potted plant acceptance:**  glm(Acceptance ~ Plant potting status * Plant state of origin, family = binomial(link = “logit”)) | | | | | | | |
| **Fixed effect term** | **Coefficient** | **St. error** | **χ^2^** | **df** | **P** | | |
| Intercept | 0.31 | 0.30 |  |  |  | | |
| Pot Status (potted) | -0.40 | 0.43 | 1.17 | 1 | 0.28 | | |
| Plant State (MD) | -4.10 | 1.06 | 62.4 | 1 | <0.001 | | |
| Interaction Term | -14.4 | 1305 | 0.63 | 1 | 0.43 | | |
| **White turtlehead acceptance:**  glm(Acceptance ~ Butterfly state of origin, family = binomial(link = “logit”)) | | | | | | | |
| **Fixed effect term** | **Coefficient** | **St. error** | **χ^2^** | **df** | **P** | | |
| Intercept | -0.20 | 0.44 |  |  |  | | |
| Butterfly State (MD) | 0.32 | 0.66 | 0.23 | 1 | 0.68 | | |

**References**

Miller JR, & Strickler KL (1984) Finding and accepting host plants. Chem. ecol.

Ins. 127-157

Singer MC (1982) Quantification of host preference by manipulation of oviposition behavior in the butterfly *Euphydryas editha*. Oecologia 52:224-229.

<https://doi.org/10.1007/BF00363841>
